# Tropism of the Chikungunya Virus

**DOI:** 10.3390/v11020175

**Published:** 2019-02-20

**Authors:** Giulia Matusali, Francesca Colavita, Licia Bordi, Eleonora Lalle, Giuseppe Ippolito, Maria R. Capobianchi, Concetta Castilletti

**Affiliations:** National Institute for Infectious Diseases “Lazzaro Spallanzani” IRCCS, 00149 Rome, Italy; licia.bordi@inmi.it (L.B.); eleonora.lalle@inmi.it (E.L.); giuseppe.ippolito@inmi.it (G.I.); maria.capobianchi@inmi.it (M.R.C.); concetta.castilletti@inmi.it (C.C.)

**Keywords:** chikungunya virus, viral tropism, arthropod vectors, animal hosts, pathogenesis, vertical transmission

## Abstract

Chikungunya virus (CHIKV) is a re-emerging mosquito-borne virus that displays a large cell and organ tropism, and causes a broad range of clinical symptoms in humans. It is maintained in nature through both urban and sylvatic cycles, involving mosquito vectors and human or vertebrate animal hosts. Although CHIKV was first isolated in 1953, its pathogenesis was only more extensively studied after its re-emergence in 2004. The unexpected spread of CHIKV to novel tropical and non-tropical areas, in some instances driven by newly competent vectors, evidenced the vulnerability of new territories to this infectious agent and its associated diseases. The comprehension of the exact CHIKV target cells and organs, mechanisms of pathogenesis, and spectrum of both competitive vectors and animal hosts is pivotal for the design of effective therapeutic strategies, vector control measures, and eradication actions.

## 1. Introduction

Chikungunya virus (CHIKV), a mosquito-borne alphavirus of the *Togaviridae* family, has caused over 70 epidemics between 1952 and 2018. It was first identified in 1952 in present day Tanzania (East Africa), and was isolated from infected patients’ sera, and from *Aedes* and *Culex* spp. mosquitoes in 1953 [1]. In the late 1950s, CHIKV was described in Uganda, the sub-Saharan region, and in Central and Southern Africa [2]. Based on the phylogenetic analysis of the CHIKV sequences from these early African outbreaks, they were grouped under the East-, Central-, and South-African lineage (ECSA) [3].

A second lineage, known as West African (WA), was retrospectively identified in mosquitoes captured in Senegal [3]. Then, the virus is thought to have moved from Africa to Asia, where CHIKV outbreaks were initially confused with dengue epidemics. Genetic analyses of CHIKV isolated from 1958 to 1973 in Asia placed them in a distinct group called the Asian lineage [3,4]. More recently (2004), a phylogenetic group, the Indian Ocean sub Lineage (IOL), originated from an ECSA clade causing, among others, a large epidemic in Réunion Island in 2005 [5].

Cases of CHIKV (IOL lineage) have been described in Europe since 2007, when an outbreak was reported in northeastern Italy, with a total of 217 cases, and the presumed index case coming back from India [6]. Since then, autochthonous cases of CHIKV fever have occurred in France, Croatia, Spain, and Italy.

In the Pacific region, CHIKV (Asian lineage) was first detected in early 2011 in New Caledonia, and later traveled to other Pacific countries, including Micronesia and French Polynesia [7,8].

In the Western Hemisphere, Asian CHIKV was initially identified in the Caribbean, precisely in Saint Martin Island, at the end of 2013, and from there it spread towards Central, North, and South America. Notably, the strains circulating in Brazil in 2014 were closely related to the ECSA isolates detected in Angola [8,9].

The most recent CHIKV outbreak was reported in Sudan, affecting seven states, with a total of 13,978 cases of chikungunya, 95% of which were from the Kassala State [10,11] (see [12] for a more extensive review on CHIKV epidemics).

## 2. Invertebrate and Vertebrate Animal Hosts

### 2.1. Invetebrate Vectors

Mosquitoes are the best-known vector of human diseases, and account for the vast majority of CHIKV transmission to humans through the urban transmission cycle (i.e., viral cycling between domestic mosquitoes and humans), as well as for the maintenance of the virus, during interepidemic periods, via the sylvatic transmission cycle (i.e., viral cycling between vectors and wild animals).

During the urban cycle, the insect species responsible for human infections are *Aedes* (*Ae*) *aegypti* and, most recently, *Ae albopictus* [13].

Nowadays, *Ae aegypti* remains the main vector for the urban cycle in Africa, the Americas, and Asia, and *Ae albopictus* is responsible for the large epidemic in the Indian Ocean Islands and for human cases in Europe, where it is the only vector present. A third species—*Ae Hensilli*—was indicated as the putative vector involved in the outbreak occurring in Micronesia in 2013 [14].

The vector competence of *Ae albopictus*, experimentally assessed in 1976 [15], was clearly demonstrated during the outbreak in the Indian Ocean region. During this epidemic, the absence of *Ae aegypti* on Reunion Island was the first clue suggesting the involvement of another vector, and on the basis of CHIKV-positive mosquito pools and competence testing, *Ae albopictus* was designated as being responsible for viral transmission, leading to the large amount of infected individuals [13]. Phylogenetic studies were carried out and a single mutation in the envelope viral gene E1 of an ECSA strain (alanine to valine at position 226, A226V E1) was considered responsible for the increased fitness of CHIKV in *Ae albopictus* and the consequent acquisition of a more effective vector competence. This mutation promoted viral replication and transmission by this highly anthropophilic mosquito [16,17], and allowed for the substantial geographic expansion of CHIKV throughout sub-Saharan Africa and Southeast Asia, and into Europe [18].

It is noteworthy that CHIKV isolated from some of the European autochthonous cases lacked the A226V substitution in E1 [19,20,21], indicating that other factors or mutations can determine the virus adaptation to *Ae albopictus*. Indeed, substitutions in the E2 and E3 genes have also been involved in the process [22,23]. These mutations are suggested to enhance the infection in the mosquito midgut, probably altering the entry process at the fusion step in the endosome [23,24].

The sylvatic transmission cycle, of known relevance in the maintenance of the virus in interepidemic periods in Africa, involves a wider range of mosquito species, including *Ae aegypti*, *Ae africanus*, *Ae luteocephalus*, *Ae furcifer*, and *Ae taylori* [25,26,27,28,29].

Moreover, several other mosquitoes have been found to be incidentally infected by CHIKV in Africa, including *Culex* spp., *Anopheles* spp., and *Mansonia* spp., nevertheless, their vector competence has not been demonstrated (for a full list of naturally infected African mosquitoes, see [30]).

Other arthropod species (i.e., non-mosquito-arthropods) do not seem to have a role in the vectorial transmission of CHIKV, but the virus has been isolated in a very low percentage of ticks collected in Senegal and the Republic of Guinea [31,32].

Data from laboratory-based competence assays further enlarge the spectrum of CHIKV potential vectors. Results from nine studies demonstrated a full competence for CHIKV transmission of mosquitoes captured in Africa (*Ae fulgens, Ae frucifer, Ae togoi, Ae triseriatus, Ae vittatus, Ae bromeliae,* and *Eretmapodites chrysogaster*), New Zealand (*Opifex fuscus*), French Polynesia (*Ae Polynesiensis*); iv) Singapore (peridomestic *Ae malayensis*), Brazil (*Haemagogus leucocelaenus* and *Ae Terrens*), and Australia (*Ae vigilax* and *Ae notoscriptus*) [33,34,35,36,37,38]. Moreover, one study demonstrated the presence of CHIKV in the saliva of *Culex gelidus* mosquitoes after intrathoracic viral inoculation [39], while other researchers observed a low rate of CHIKV transmission for *Ae koreicus*, a mosquito new to Europe [40]. It is noteworthy that mosquito vectors display different degrees of vector competence for different CHIKV isolates, and the nsP3 viral protein seems to be a determinant of vector specificity [41].

CHIKV is horizontally transmitted to vectors during a blood meal on a viremic host with CHIKV viral titers ranging from 10^3^–10^5^ plaque-forming units per ml (PFU/mL) [42].

Once ingested, the virus reaches the midgut, where it penetrates the epithelium, probably via clathrin-mediated endocytosis [43], and it replicates.

There are two mechanisms proposed for the dissemination of CHIKV from the midgut to secondary organs, as follows: (i) newly formed chikungunya virions accumulate at the level of the midgut basal lamina (BL), and pass to secondary organs thanks to collagenases mediated BL remodeling [44,45,46]; (ii) virions enter the tracheal cells’ system surrounding the midgut and travel to secondary organs, a mechanism described for other arboviruses [47,48,49].

Three to seven days post infection, whether through the direct passage of the BL or the infection of the tracheal system, the virus spreads to distant anatomical districts (i.e., from the abdomen to the head), eventually reaching the salivary glands [33,50].

CHIKV replicates and is stored in the acinar cells of the *Aedes* mosquito salivary glands, where it apparently does not cause a cytopathic effect [51,52,53,54].

**Figure 1:**
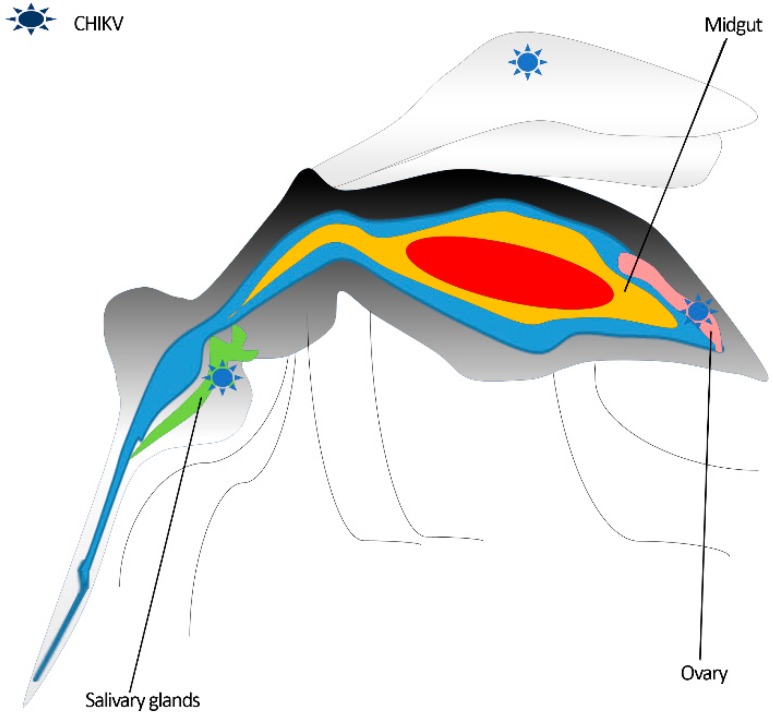
Sites of chikungunya virus replication in the *Aedes* mosquito vector.

CHIKV infection of the fat bodies of the *Aedes* species, described for other arboviruses (e.g., dengue virus 2 (DENV 2) and Zika virus (ZIKV)) [49,55], is currently undetermined. On the contrary, viral isolation from the legs and wings indicate the presence of CHIKV in these districts [56] (see Figure 1 for CHIKV mosquito tissue tropism).

Interestingly, the ovary is also infected by CHIKV, and replication at this site can determine the vertical transmission [57,58,59,60,61]. However, this transmission route seems to be rare [61], and some authors argue against the infectivity of the virus persisting in eggs [62]. The transovarial transmission of CHIKV through eggs potentially leads to virus survival in adverse environmental condition (eggs resist desiccation during the dry season) [63,64]. On the other hand, CHIKV seems to perturb the egg-laying pathway—resulting in a lower number of eggs—and survival rate of infected mosquitoes [57].

### 2.2. Vertebrate Animal Hosts Spectrum

Non-human vertebrate hosts have a prominent role in the maintenance of arboviruses in the enzootic cycle of infection. They constitute a viral reservoir from which the spillover to the human population may occur.

In Africa, during inter-epidemic periods, CHIKV is believed to be preserved via a sylvatic transmission cycle involving arboreal mosquitoes and wild primates.

This is in contrast to Asia, where the virus principally cycles between the two main vectors (i.e., *Ae aegypti* and *Ae albopictus*) and humans [13]. The role of vertebrate animals of new endemic regions (i.e., the Americas and the Pacific) and Europe in the amplification and survival of CHIKV has been poorly investigated to date. Indeed, the majority of studies evaluating the natural infection of non-human-vertebrate hosts have been realized in Africa (12 studies) (reviewed in [65] and [66,67,68]), and evidenced the prominent role of non-human primates (NHPs) in sylvatic transmission (Table 1).

In the early studies carried out in Senegal, CHIKV was isolated from non-human primates (NHPs) (i.e., *Cercopithecus aethiops, Galago senegalensis, Papio papio, Erythrocebus patas,* and *Chlorocebus sabaeus*), palm squirrel (*Xerus erythropus*), and bats of the *Scotophillus* species [27,31]. Seroprevalence studies enlarged the range of naturally infected mammals. Specific antibodies have been detected in the sera of several primates (*Cercopithecus aethiops, Cercopithecus Ascanius, Cercopithecus mitis, Papio cynocephalus, Papio papio, Papio ursinus, Mandrillus sphinx, Galago senegalensis, Erythrocebus patas, Eulemur fulvus,* and *Macaca fascicularis*), and with a low prevalence in one out of the 10 rodent species tested (ship rats, i.e., *Rattus rattus*) (reviewed in [65] and [66,67,69]).

Notably, neutralizing antibodies against CHIKV were measured in one forest buffalo (*Syncerus caffer nanus*) and one elephant (*Loxodonta africana*) from the Congo basin [70]. In contrast, negative results were obtained when testing domestic and farm animals, such as cats, dogs, cattle, goats, horses, sheep, pigs, and poultry [69,71,72]. To understand whether a sylvatic cycle can be established outside Africa, six studies investigated Asian NHPs’ natural infection, founding CHIKV-specific antibodies in macaques (*Macaca fascicularis* and *Macaca nemestrina*) [73,74,75,76,77,78], and only one report was published on the infection and seroprevalence of CHIKV in American NHPs [79]. The American study showed low seroprevalence and antibody titers with negative RT-PCR results in 11 monkeys of five different species (*Sapajus flavius, Sapajus robustu, Sapajus xanthosternos, Ateles marginatus,* and *Callithrix jacchusurban*) in urban and peri-urban neotropical NHPs sampled in Brazil.

Given the results of non-human-vertebrates’ natural infection, experimental investigations on host susceptibility to CHIKV were mostly conducted in NHPs and rodents, with the double aim of studying their potential role as a reservoir, and of establishing animal models of infection (reviewed in [65]). These studies show the higher susceptibility of infant or immunodeficient mice (as compared to adult mice), the infection of Rhesus and Cynomolgus macaques (i.e., *Macaca mulatta* and *Macaca fascicularis*), widely used experimental models, and of bats—a well-known reservoir involved in the spreading of emerging viruses.

Two trials of experimental viral inoculation, using two ECSA strains (one isolated from a patient in South Africa in the 1970s, the other from Comoros Island mosquitoes in 2005), have been recently performed to identify potentially competent hosts among domestic and wild animals common to North America [80,81]. The authors tested nine avian, twelve mammalian, three amphibian, and seven reptilian species in order to assess the onset of viremia and seroconversion upon CHIKV infection. The animals with detectable CHIKV viremia were some mammals (i.e., hamsters, C57BL/6 mice, and big brown bats), amphibians (Leopard frog and Texas toad), and reptiles (Ball python, Burmese python, Garter snake, Green iguana, Red-eared slider), and seroconversion occurred in all of the viremic reptiles, in Texas toads, and in bats. Moreover, a neutralizing antibody titer was measured in some of the non-viremic species, including domestic/farm/wild mammals (dog, pig, horse, calf, goat, armadillo, and raccoon), and a small proportion of birds. None of the tested animals developed clinical signs [81].

The investigations described suggest that, together with NHPs, some bats and rodents, as well as ectothermic animals, could serve as virus-amplifying hosts, while lessening the likelihood that numerous birds and non-primate mammals are competent reservoirs for CHIKV.

## 3. CHIKV Cellular Receptors

Viral tropism is defined as the capacity of a virus to infect specific cells, tissues, and species. It depends on both viral and cellular characteristics—with the presence of the cognate receptors for viral attachment molecules being of undeniable importance [83]. The CHIKV protein that facilitates cell binding is the E2 glycoprotein, while the related cellular receptor/s crucial for CHIKV entry has/have yet to be recognized [83]. Given the large tropism of CHIKV, its putative receptor/s is likely ubiquitously expressed among species and cell types [83].

The first molecule indicated as a CHIKV receptor in 2012 was Prohibitin 1 (PHB1), a multifunctional membrane protein expressed by numerous cell types [84]. In microglial cells, PHB1 was shown to co-immunoprecipitate with the CHIKV E2 protein, and the number of infected cells was significantly lowered when using anti-PHB1 blocking antibodies (a reduction of 20% to 40% compared with the untreated control) or upon PHB1 silencing (reduction of approximately 30%). In the presence of blocking antibodies, the viral production was also significantly reduced [84]. The same group later confirmed the role of PHB1 in viral entry by pre-incubating HEK-293T cells with flavaglines (prohubitin ligands) before CHIKV infection. This pre-treatment reduced both the percentage of infected cells (20% to 40% reduction) and the viral production (approximately 40% reduction) [85]. In 2018, Michael Diamond and colleagues, through a genome-wide screening for the cellular factors required for CHIKV entry, identified the cell adhesion molecule Mxra8. They demonstrated that blocking this molecule prior to CHIKV infection significantly reduced the number of human primary synovial fibroblasts (50% reduction .ca), skeletal muscle cells (70% reduction .ca), osteoblasts (70% reduction .ca), and chondrocytes (70% reduction .ca) [86]. In a mouse model of in vivo CHIKV infection, the administration of the Mxr8a-Fc protein or blocking antibodies reduced both foot swelling and CHIKV infection with a differential efficiency, based on treatment kinetics [86]. Nevertheless, the lack of Mxra8 on the membranes of some CHIKV target cells (e.g., keratinocytes) indicates that this factor can be dispensable.

Two other molecules, T-cell immunoglobulin and mucin domain-1 (TIM-1), recently involved in ZIKV entry, and Glycosaminoglycans (GAGs), important for the efficient infection of alphaviruses (i.e., Eastern equine encephalitis virus (EEEV) and Venezuelan equine encephalitis virus (VEEV)), have been implicated in CHIKV attachment (reviewed in [83]) to human cells, while HSC70 and ATP synthase beta subunit have been suggested as CHIKV binding factors in mosquito cells [87,88].

Overall, a plethora of cell membrane molecules have been indicated as putative CHIKV binding/entry factors, but no definitive identification has been established.

## 4. Human Infection

Following an infected mosquito bite, CHIKV is introduced into the human skin and into the bloodstream, causing high viremia, and when it reaches the target organs, it gives rise to pathological signs. Infection in humans usually manifests as fever, myalgia, and arthralgia, and in a small percentage of individuals, it gives rise to a range of so called “atypical signs” (defined as symptoms other than fever, myalgia, and arthralgia). The virus also replicates in lymphoid organs either before (axillary lymph nodes) and/or after the passage in the blood stream (lymph nodes and spleen). Moreover, non-arthropod-borne routes of transmission have been described (vertical transmission) or suspected (sexual transmission).

### 4.1. Stages and Clinical Signs of Chikungunya Infection

One major challenge for clinicians, nowadays, is to differentiate the clinical signs of chikungunya from those of Zika and dengue fever, especially when these viruses are co-circulating. Unlike ZIKV and DENV, whose infections are asymptomatic in about 62% and 75% of individuals, respectively [89,90], CHIKV causes symptoms in a high majority (72% to 95%) of infected people [91,92]. CHIKV infects humans through the bite of *Aedes* spp. mosquito vectors, and causes the chikungunya virus disease (CHIKD), characterized, in the acute phase, by an abrupt onset of febrile illness (>39 °C in 92% of patients), frequently accompanied by joint pain (arthralgia) (87% of patients) [93].

Other common signs and symptoms include headache; fatigue; muscle pain (myalgia); distal joint swelling; cutaneous manifestations, such as macular or maculopapular rash; and gastrointestinal tract affections, such as nausea, vomiting, and abdominal pain [93,94]. Less frequently, ocular manifestations can occur, and range from photophobia to retro-orbital pain and conjunctivitis [95].

Chikungunya chronic disease is characterized by the persistence or relapse of joint pain, which was first reported in 1979 [96], and has been extensively described during recent/previous epidemics [97]. Joint pain may present for several weeks to months, and may mimic rheumatoid arthritis.

Rodriguez-Morales and coworkers systematically reviewed the data on post-chikungunya infection with chronic inflammatory arthritis in reports published from 2007 to 2015, and calculated that among the 5702 patients who were followed-up for more than 18 months, the pooled prevalence of chronic inflammatory rheumatism (CIR) associated to CHIKV infection was 40.2% [98].

A growing number of atypical signs and symptoms have been described during the last outbreaks. These include both mild and severe syndromes affecting several organs.

Economopoulou and colleagues reported that during the large 2005–2006 CHIKV outbreak on the Reunion Island, the proportion of atypical cases (defined as patients with laboratory confirmed CHIKV infection and symptoms other than fever and arthralgia) was 0.3 %; 36% of these atypical cases presented as severe cases, with a mortality of 29% (an overall case-fatality rate of 10. 7%) [99].

Cohort studies and case reports showed that the incidence rate of atypical, severe, and fatal cases increases with age in patients over 65 years old [99], and the presence of underlying medical conditions (i.e., preexisting respiratory or cardiovascular diseases and hypertension) is correlated to the occurrence of severe manifestations [100,101,102].

Unusual manifestations of chikungunya infection comprise cardiovascular, renal, cutaneous, ocular, hepatic, and respiratory syndromes [103,104,105,106].

Among the severe forms of CHIKD, it is important to mention the neurological complications (approximately 0.1% of CHIKV infection develops neurological disorders), most commonly encephalitis and encephalopathy, prompted the investigation of CHIKV tropism for the central nervous system (CNS) [107]. Acute flaccid paralysis and meningoencephalitis have also been described [99,108,109]. Moreover, Guillain–Barré syndrome (GBS), an acute inflammatory demyelinating polyneuropathy that usually occurs after infection by a variety of agents, including arboviruses, has been associated to CHIKV infection [107,110,111,112]. The tropism of CHIKV in the nervous system will be further detailed in Section 4.3.3.

The extensive array of clinical signs described during the acute and chronic phases of infection suggests a broad tissue tropism.

### 4.2. Host Pathogen Interaction

Upon infection, CHIKV, like other alphaviruses, strongly induces Type I interferons (IFN-I; IFNα and IFNβ) production [113], and the effect of these cytokines in controlling CHIKV replication has been extensively investigated.

IFN-I and IFN stimulated genes are key factors of the innate antiviral immunity [114], and the role of IFN-I response in the context of CHIKV infection is evidenced by the full susceptibility to severe CHIKV infection of IFN-type-I receptors deficient (IFNAR -/-), in contrast to adult wild-type (wt) mice [115]. In vitro, CHIKV does not directly stimulate IFN-I production in immune cells. In contrast, infected non-hematopoietic cells (fibroblasts) sense viral RNA in a Cardif-dependent manner, and participate in the control of infection through the production of IFN-I [115].

In wt mice, upon intradermal inoculation, the viral replication was controlled by the action of IFN-I derived from fibroblasts, and the peak of IFN correlated with the decline in the CHIKV load. This prompt inhibition avoided CHIKV dissemination [115].

In infected patients and NHPs, the viral load in blood positively correlates with IFN-α production [115,116], but the IFN-α burden is nevertheless not sufficient to halt the spread to target organs. This can be in part due to the counteraction of CHIKV proteins. Indeed, the viral nonstructural protein 2 (nsP2) has been shown to inhibit IFN stimulated JAK-STAT signaling [117], and the nonstructural protein 1 (nsP1) contrast viral restriction mediated by the IFN stimulated gene product BST-2/Tetherin [118]. Whether IFN-type-I overproduction may result in the exacerbation of clinical symptoms, as suggested in a case report [119], should be further investigated.

Another mechanism of evasion from the immune control is mediated through the mobilization of the apoptotic machinery. Apoptosis is a defense mechanism to limit virus production and spread [120], but in vitro experiments demonstrated that CHIKV is able to hide in apoptotic blebs and infect bystander cells [121].

Viral replication is also accompanied by upregulated levels of inflammatory cytokines (such as IL-6, MCP1 (CCL2), IP-10 (CXCL10), MIG (CXCL9), IL1β, and tumor necrosis factor (TNFα)) in the plasma of acutely infected CHIKV patients and NHPs [116,122,123,124,125,126,127]. Notably, IL-6, IL-1b, and TNFα are pyrogenic cytokines contributing to fever [128], and are implicated in persistence and tissue destruction during arthralgia [122,128,129]. IL-6 is also a marker of poor prognosis, together with IL1β and RANTES [128].

A plethora of other cytokines/chemokines have been indicated as forming a generic acute CHIKV signature in a meta-analysis conducted in all patient cohorts from all around the world [130]; these include pro-inflammatory, anti-inflammatory, chemoattractant, and growth factors [130].

Another factor involved in the modulation of CHIKV pathogenesis is the interferon regulatory factor 1 (IRF-1), which in an IFN-independent manner, restricts CHIKV replication in muscle cells and contrasts the infiltration of neutrophils and eosinophils in the joint tissues of mice [131].

Monocytes/macrophages also have a role in CHIKV pathogenesis, both secreting inflammatory mediators, migrating to distal districts, and being the site of acute and persistent infection, as more extensively described next in this review (see Section 4.3.1, 4.3.2, and 4.3.4).

Natural killer (NK) cells showed activated profiles during acute CHIKV [132], and NK cells’ infiltration in the synovial tissue is suggested to participate in the pathogenesis of CHIKV-driven arthralgia [129].

Adaptive immunity is mediated by B and T cells. CHIKV induces a robust humoral response leading to the production of anti-CHIKV IgM and IgG [133]. The importance of the antibody response was corroborated by passive immunization investigations and vaccine studies (see Section 4.4). The importance of T and B cells has also been demonstrated in Rag1 knockout mice (which lack T and B cells), as these animals develop higher viral levels and disease severity compared with wt mice [134]. CD4+T cells are also involved in inflammation and the development of joint swelling [134,135]. CD8+T cells are strongly induced by vaccination in animal models, leading to an efficient cellular cytotoxic immune response (together with humoral immunity) [136,137]. Interestingly, age-related vulnerability as a result of reduced IFN-I, and T and B cell immunity has been demonstrated in NHP models [138].

The role of immune mediators at specific sites of replication will be further reviewed in the next sections.

### 4.3. Cellular and Tissue Tropism in Human Infections

#### 4.3.1. Infection of Skin and Blood Cells

In human skin, the first human organs supporting viral replication upon a mosquito bite, the dermal fibroblasts, constitute the main site of viral amplification, as demonstrated in vitro [139,140,141], in mice models (IFN-type-I receptors deficient or neonates animals), and by the detection of viral antigens in a skin biopsy from a neonatal fatal case [142]. In these cells, CHIKV infection determines a strong antiviral IFN-type-I (IFN-I) response, alongside the production of pro-inflammatory cytokines. The induction of many antiviral genes (e.g., viral sensors, IFN-I receptors, and IFN stimulated genes (ISGs)) is however contrasted by components of *Aedes* mosquito saliva, thus favoring viral replication [141].

The same pro-viral effect of the mosquito salivary glands’ extract was described upon in vitro infection of skin keratinocyte with an ECSA strain [143]. Contrasting results were obtained by another research group, who used IOL CHIKV strains encoding GFP or mCherry reporter genes to infect keratinocytes [144]. In their experimental conditions, the viral early steps of replication (binding and entry) were completed, while non-structural proteins and genomic RNA were poorly expressed, probably because of an IFN type I, II, and III mediated restriction. Notably, viral infection resulted in a transient induction of type I and II IFN genes, and a continuing increase of IFN-III mRNA (IFN type III being a cytokine preferentially expressed by epithelial cells). The susceptibility of primary human keratinocytes to CHIKV was recently confirmed by Zhang et al. [86], where the virus entered the cells in a Mxra8 independent manner.

In mice, the infection of keratinocytes was observed in interferon response factor 3 and 7 deficient (IRF3-/IRF7-), but not in wt animals [145]. As IRF-3 and -7 are regulatorx of type-I and -III IFNs [146,147], this mouse model supports a role for these antiviral pathways in CHIKV restriction.

**Figure 2:**
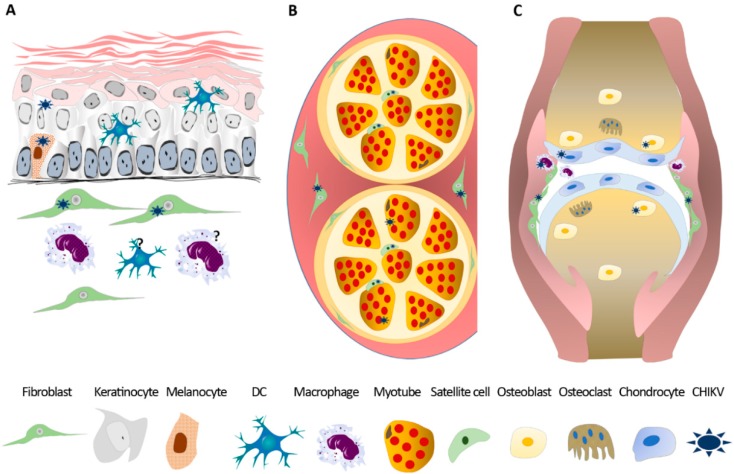
Examples of tissue and cell tropism of chikungunya virus. Chikungunya virus (CHIKV) infected cells in skin (**A**); skeletal muscle (**B**); joint and bone (**C**). DC—dendritic cell.

Along with the dermal fibroblasts and keratinocytes, melanocytes are also permissive for CHIKV [148], while the infection of dermal macrophages is only assumed by the susceptibility of the blood derived and resident macrophages of other tissues (see Figure 2 for skin tropism).

The route from the skin to other anatomical districts has been characterized in NHP models, where CHIKV travels from the site of infection (i.e., the skin) into the draining axillary lymph nodes, and then causes viremia [116].

Viral replication in human blood was first suggested by strong viremia (the blood viral load can reach up to 10^9^ RNA copies/ml) [149], but one pioneering study showed that the only in vitro infected lymphoid cells producing viral particles were primary monocyte-derived-macrophages [140]. Since then, CHIKV tropism for blood cells has been more extensively studied, and the active infection of human blood monocytes and, to a lesser extent, of B lymphocytes and plasmacytoid dendritic cells (pDCs), was demonstrated in peripheral blood mononuclear cells (PBMCs) derived from acutely infected patients, and upon in vitro infection [124,150,151]. Viral replication was accompanied by upregulated levels of immune mediators (e.g., IFNα, IL-6, MCP1 (CCL2), IP-10 (CXCL10), MIP-1β (CCL4), and IL1Rα, MIG (CXCL9), IL1β, and TNFα) [150] and high levels of inflammatory cytokines (such as IFNα, IL-6, MCP1 (CCL2), IP-10 (CXCL10), MIG (CXCL9), IL1β, and TNFα) in the plasma of acutely infected CHIKV patients was found to correlate with high viral titers or disease severity [122,123,124,125,126,127]. Similar findings in NHPs further validate these models of CHIKV infection/pathogenesis [116].

Interestingly, viral replication dynamics in PBMCs experimentally infected with CHIKV and DENV resulted in a significant reduction of CHIKV progeny and a moderate enhancement of DENV production. Co-infection also led to higher levels of IL-6 and TNFα, implicated in the pathogenesis of the two arboviral diseases [152]. The effect of co-infections on CHIKV tropism and pathogenesis is ill-defined and should be further investigated both in humans and animal models.

Finally, CHIKV can also associate to red blood cells and platelets, a notion that should be considered for blood transfusion safety [153,154].

#### 4.3.2. Infection of Muscle, Joint, and Bone

During the acute and subacute phase, CHIKV reaches the muscle and joint compartments where it gives rise to the two main symptoms associated to this arbovirus—myalgia and arthralgia. During the Indian Ocean Islands outbreak, human muscle biopsies derived from two patients (one acutely infected and the other relapsing) were stained positive for CHIKV antigens in fusiform curved shape cells co-labelling with specific markers (i.e., laminin or neural cell adhesion molecule) of satellite cells [155]. The same study analyzed the myo-tropism in vitro, showing that, unlike satellite cells, differentiated myotubes were spared from infection. Nevertheless, primary human myoblasts were found to be permissive to CHIKV in a more recent study [156], and skeletal muscle fibroblasts were stained positive for viral antigens in a fatal neonatal case [142].

The ability of CHIKV to infect progenitor cells and to induce skeletal muscle necrosis may explain the CHIKV-induced myopathies, and implicate the muscle as a site of viral persistence.

The other distinctive CHIKV clinical syndrome is arthralgia, which is generally of symmetric type, with the distal synovial joints more commonly affected than the proximal [129].

CHIKV RNA and proteins have been identified in the synovial tissues and fluids during acute and persistent arthralgia [129,149,155,157,158], and both synovial fibroblasts and macrophages are susceptible to CHIKV [86,129,142,159]. The histological analysis of a chronic patient’s biopsy showed the active replication of CHIKV in joint macrophages’ infiltration of activated NK cells and T cells (mainly CD4), and extensive apoptosis, while the analysis of the protein content of the synovial fluid revealed high levels of matrix metalloproteinase MMP2 (involved in tissue remodeling), CCL2, IL8 (monocytes and neutrophils chemo-attractive cytokines), and IL6 (multifunctional cytokine involved in inflammation) [129]. These data show that CHIKV-infected macrophages are a preferential site of viral persistence, in which the active replication of the virus take place and contributes to chronic symptoms.

Together with joint inflammation, cartilage degradation also takes place in CHIKV infected individuals. In humans, the presence of elevated levels of cartilage bioproducts in urine (i.e., proline, hydroxyproline, and mucopolysaccarides) [160], and the low levels of the plasmatic of hepatocyte growth factor (HGF; hormone facilitating cartilage repairs) in chronic patients [122], are indicative of connective tissue alteration and cartilage damage. In mouse models of CHIKV-induced arthritis, the destruction of cartilage and the formation of large areas of collagenosis and fibrosis were observed [125,161,162]. A direct effect of CHIKV on cartilage is suggested by viral replication in human primary chondrocytes [86] and in CHIKV inoculated immunodeficient (IRF3/7^−/−^) mice [145].

Bone loss is another hallmark of CHIKV arthritis, and osteoblasts are a site of CHIKV replication and persistence [86,163,164,165]. Ex vivo infection of human osteoblasts induced the secretion of IL6 and receptor activator of nuclear factor-kB ligand (RANKL), and a concomitant decrease in the osteoproteogerin (OPG) content in culture supernatants. This perturbation in the RANKL/OPG ratio has been evidenced in infected murine joints and in patients’ sera [165]. The dysregulation in the RANKL/OPG ratio in CHIKV-infected joints, and the upregulation of monocyte chemoattractant proteins observed in mice joints (CCL2, CCL7, and CCL8) and in the serum of CHIKV patients (CCL2) [122,123,129,165] favors an osteoclastogenic and chemo-attractive microenvironment. This was confirmed by the local infiltration of monocytes in CHIKV-infected animals [125,166,167], and by the ability of supernatants of CHIKV infected synovial fibroblasts to induce migration and differentiation into the osteoclast-like cells of primary human monocytes [159]. Importantly, the inhibition of monocytes/macrophages recruitment or macrophages depletion attenuated arthritis and joint swelling in mouse models [165,167], but resulted in prolonged viremia, suggesting that macrophages are also required for the clearance of the virus [167] (see Figure 2 for CHIKV tropism in muscle, joint, and bone).

#### 4.3.3. Infection of the Nervous System

CHIKV is not considered to be a true neurotropic virus (i.e., able to invade the neural tissue and replicate in the neurons), however cases of neurological manifestations have been reported in the outbreaks that have occurred since the ones in Asia in the 1960s and 1970s [168,169,170]. Upon the re-emergence of CHIKV in the Indian Ocean in 2005, a growing number of cases of neurological complications (e.g., meningitis, encephalitis, febrile seizures, Guillain Barré syndrome, and acute flaccid paralysis) associated to this arboviral infection were recognized, and the tropism of this agent for the nervous system was better characterized [107,169]. In patients presenting with neurological disease, the virus was frequently evidenced in the cerebrospinal fluid (CSF) by viral isolation or by RT-PCR (systematically reviewed in [107]), while in the few brain autopsies analyzed for histopathological studies, the presence of CHIKV antigens was not evaluated [171]. As a consequence, the target cells of CHIKV in the human brain remain unknown. Nevertheless, the in vitro infection of human cells demonstrated the susceptibility of neuroblastoma cells (neural crest derived cells) [172,173], and of glial cells, such as astrocytes [174,175], and microglial cells [176,177] to CHIKV. The apoptosis observed in CHIKV infected neuroblastoma cells and astrocytes suggests a direct implication of viral infection in disease pathogenesis. In experimentally infected mice, the virus preferentially infects astrocytes, and, to a lesser extent, neurons, while the infection of oligodendrocytes was evidenced in vitro [178,179,180]. In NHPs, CHIKV RNA was detected in the brain during the acute phase, and was shown to persist in the CSF [166].

Finally, the endothelial brain cells of human and animal origin can, in some instances, produce CHIKV infective particles [140,181,182].

The mechanisms by which CHIKV affects the human nervous system remain ill defined. It is still unclear whether it acts directly by targeting neurons and glial cells, or indirectly by triggering immune mediated effects (i.e., through the upregulation of inflammatory and antiviral cytokines).

In the nervous system, the eye may be injured in CHIKV patients. Ocular diseases present mainly as retro-orbital pain or conjunctivitis at the time of CHIKV systemic illness, and predominantly as uveitis, retinitis, or optic neuritis in later phases and recurrences [107,183,184,185,186].

The presence of viral RNA in ocular fluid was reported three times [187,188,189], and in a laboratory screening of CHIKV infection of corneal donors, infectious viral particles and RNA were detected in the eye tissue from four donors [189]. The characterization of CHIKV positive cells in the human eye revealed the infection of fibroblasts of the corneal and scleral stroma, corneal endothelium, ciliary body, iris, and between ocular muscle fibers [189]. Based on these results, the risk of CHIKV iatrogenic transmission through corneal transplantation must be taken into account.

#### 4.3.4. Infection of other Target Organs

The wide range of secondary organs targeted by CHIKV may explain the rare consequences of chikungunya infection, and the clinical manifestations observed in patients (i.e., renal, respiratory, hepatic, cardiac, and neural syndromes) [103,104].

A relevant model of infection showing viremia and clinical signs similar to those seen in humans was developed by Labadie and coworkers [166], who inoculated a CHIKV IOL isolate into 15 cynomolgus macaques (*Macaca fascicularis*). High titers of viral RNA were measured in lymphoid organs as well as in the liver, where viral antigens localized in macrophages, DCs, and endothelial cells. The acute phase was also characterized by strong mononuclear cells’ infiltrations in the spleen and lymph nodes [166]. The spleen, liver, and heart have been found to be positive for CHIKV in another NHP model [190], and viral RNA has been detected in the liver of a human fatal case [191].

Moreover, in vitro and in vivo findings suggest that kidneys and lungs are also targeted by CHIKV [140,177,190,192]. Both human and animal derived kidney epithelial and lung epithelial and fibroblasts cell lines are indeed susceptible to CHIKV, and are widely used for viral assays [140,177,192]. Nevertheless, the exact target cells of CHIKV in human secondary target organs (e.g., the kidney, liver, heart, lungs, etc.) and its ability to persist in such anatomical sites remains unclear.

It is noteworthy that, when speaking of viral secondary sites of replication, viral RNA can, in some instances, be detected in body fluid specimens such as saliva and urine. In the saliva, CHIKV seems to originate from oral bleeding [193], while whether viral RNA in urine is derived from local renal replication is unknown [194,195]. During the Italian outbreak in 2017, we screened 1414 serum specimens for the presence of CHIKV RNA, and 196 of these resulted positive. For some viremic patients, body fluids other than serum became available for further diagnostic study, and viral RNA was detected in one saliva, one urine, and one cervico-vaginal fluid (CVF) specimen (unpublished data) (Table 2).

#### 4.3.5. Target Organs upon Non-Arthropod-Borne Transmission

Cases of mother to child CHIKV transmission have been reported, and a recent systematic review and meta-analysis evaluated the risk of this secondary route of viral spread [196]. Across the cohorts examined in this review, the overall risk for mother to child transmission was 15.5% (206/1331), with risk for symptomatic neonatal disease as 50.0% among intrapartum, and 0% among antepartum/peripartum maternal infections. Symptomatic newborns develop a wide range of clinical signs, including fever; arthralgia; irritability; poor feeding; rashes; and, occasionally, multiple organ involvement, meningoencephalitis, and long term neurodevelopmental delays [196]. In a cohort study, delivery via caesarean section appeared to have no influence on the transmission risk, supporting the notion of the trans-placental transmission of CHIKV, rather than exposure in the birth canal [197]. Notably, three cases of early maternal–fetal transmission (<16 weeks’ gestational age) occurred during the outbreak in Reunion Island, which reportedly described the presence of viral RNA in the amniotic fluid (three fetuses), in the placentas (two fetuses), and in the brains (two fetuses) of aborted fetuses [198]. In a cohort study from the same epidemics, CHIKV RNA was again detected in the amniotic fluid (4/23 samples) and placental tissue (19/462) [199], and a fatal Brazilian fetal case of the Zika virus and CHIKV co-infection presented with CHIV RNA in the placenta [200].

Some authors argue against the placental tropism of CHIKV, as the replicating virus has never been isolated from infected placental tissues; and human syncytiotrophoblastic cell lines are refractory to infection in vitro [142,201].

Nowadays, the hypothesized mechanism of CHIKV vertical transmission is the passage of the virus from the maternal bloodstream through breaches in the placenta, a pathway supported by a study in an IFN-alpha-receptor knock-out mouse model [142]. However, recent research showed in vitro CHIKV replication in human fetal membranes (from the second trimester), but not in maternal-derived decidua or fetal-derived chorionic villi [202]. Further studies are undeniably necessary in order to dissect the mechanisms and to reveal the risk factors of CHIKV mother to child transmission.

Another non-arthropod-borne route under investigation is the sexually driven spreading. Indeed, since the discovery of ZIKV sexual transmission, great efforts have been made to understand whether other arboviruses are able to infect the genital tract, and whether they can be passed through sexual intercourse. CHIKV RNA was detected in the seminal fluid of one patient who was co-infected with DENV3. In this patient, the semen remained positive one month from the onset of symptoms [203].

To our knowledge, together with the detection of CHIKV RNA in the CVF, which we show in Table 2, these are the only reported cases of CHIKV detection in genital fluids, and the tropism of the virus for the genital tract, as well as its potential to be transmitted through male or female genital secretions, should be further investigated.

### 4.4. Preventive and Therapeutic Strategies

Despite the fact that CHIKV causes disease around the globe, with a significant impact on the quality of patients’ life as well as the relevant economic burden, no specific anti-viral drugs or/and licensed vaccines are available, to date. Treatment is entirely symptomatic, and only palliative care can be administered (analgesics, antipyretics, non-steroidal anti-inflammatory drugs (NSAIDs), and systemic glucocorticoids in the case of severe and persistent joint pain) [204]. CHIKV infection may not always present specific manifestations, and it may co-exist with other infectious diseases, like dengue or Zika fever [205]. Thus, careful clinical management in the absence of laboratory confirmation is crucial. According to the WHO guidelines, paracetamol is the first-line analgesic treatment, and aspirin or other NSAIDs should be avoided, because of their adverse effects and the increased risk of bleeding manifestations in cases of DENV (co-)infection [206].

To find an effective treatment for CHIKV, numerous antivirals have been investigated. In vitro and in animal model studies showed that licensed antiviral compounds for other RNA viruses, such as Ribavirin, Favipiravir, IFN-α, and Sofosbuvir, may have a positive effect in controlling CHIKV replication [8,207,208,209,210]. Chloroquine (CQ), a common antimalarial medicine, was widely used to treat rheumatoid arthritis-like symptoms, following CHIKV infection during the Reunion Island outbreak [204,211]. Although CQ inhibits CHIKV replication in vitro [212,213], it showed no or a moderate effect on CHIKV acute and chronic symptoms in humans [211,214], and in NHPs, it even determined enhanced CHIKV replication and delayed cellular and humoral response [214].

Immunotherapy using anti-CHIKV monoclonal antibodies represents a different approach for therapeutic and post-exposure interventions, and studies conducted on mice models evidenced good protection, even when administered at later time points after infection [208,215].

Because of the limited diversity between CHIKV strains, vaccine strategy represents the best preventive measure to limit the spread of infection and protect people from the disease. Despite the fact that none have been approved, several vaccine candidates have been investigated in preclinical and clinical studies. From the first vaccines prepared using the formalin-inactivated approach, various live-attenuated candidates have been developed and tested [216]. TSI-GSD-218 was the first live-attenuated CHIKV vaccine reported in trial, and was made using CHIK 181/clone 25 serially passed in cell cultured; it was shown to provide effective and lasting immunity, but in phase II, it caused mild arthralgia in 8% of the vaccinees [208]. Thereafter, the research moved towards other methodologies in order to ensure optimal safety and higher levels of protection and immunogenicity [208]. Examples are the CHIKV/IRES vaccine [217], and those preparations containing alphavirus chimeras [218] or modifications in the viral genome, such deletions [217] or codon alteration [219]. New promising candidates are the replication-deficient viral-like particles (VLP) vaccines (based on cDNA expression plasmid transfected into human cells, and containing CHIKV structural proteins), and the viral vectored vaccines (VVV), which use recombinant replication-deficient viruses as antigen delivery vectors (i.e., the measles virus (MV-CHIV) and adenovirus derived from a chimpanzee (ChAdOx)) [216]. The VRC-CHKVLP059-00-VP VLP vaccine entered a phase II trial in 2015; MV-CHIKV VVV completed a phase II trial with a good level safety and immunogenicity; the ChAdOx1-Chik vaccine is in a phase I trial, and the experimentation is ongoing [216].

In waiting for the final licensed vaccines and drugs to be used especially in endemic areas and risk population, or in case of epidemics, a great contribution of public health value is recognized in vector-control tools (i.e., insecticides, larvicides, traps, and repellents for personal protection) and integrated alternative strategies, which are under evaluation, and mainly aim to suppress vector mosquito populations and avoid the risk of resistance to chemical interventions (i.e., sterile insect technique (SIT), release of insects with dominant lethality (RIDL), and Wolbachia-infected males) [220].

## 5. Conclusions

Before 2004, CHIKV caused small outbreaks in confined regions within Africa and Asia. Then, the virus started to cause large outbreaks, and spread to novel areas, including Europe, the Americas, and the Pacific Islands. Notably, no specific antiviral treatment is available yet, and the case management of CHIKD patients is based on supportive care.

While early research studies are mainly focused on the identification of viral vectors and natural reservoirs, the understanding of the pathogenesis and the biology of the virus greatly improved since its re-emergence.

The clinical signs of CHIKD have been better characterized, and several syndromes have been newly associated to CHIKV infection.

The expansion to novel regions and the drastic increase of clinical cases prompted the scientific community to actively investigate the pathogenic and ecological aspect, including the tissue and cell tropism of the virus, the immune response of the host, the mechanisms of viral persistence, the competence of arthropod vectors, the spectrum of other potential animal hosts, and the urban and sylvatic cycle of CHIKV.

Studies on the viral tropism demonstrated preferential replication in fibroblasts and monocytes/macrophages, which, nevertheless, are not the only viral targets. Indeed, a number of other cells, including epithelial, endothelial, and muscular cells, are permissive to CHIKV, as established from in vitro studies and supported by ex vivo findings. Nevertheless, the exact cellular receptors for CHIKV need to be definitively established.

At the tissue level, CHIKV is amplified at the site of infection, the skin, and in the blood monocytes, which then reaches both the lymphoid and non-lymphoid organs, including the skeletal muscle and the synovial joints. At these sites, CHIKV may persist for several months, giving rise to chronic arthralgia, a hallmark of CHIKD.

The mechanisms leading to atypical diseases need to be further investigated, as it is the case of neurological manifestations, where it could be the result of a direct infection of the CNS or of the inflammatory pathways triggered by the virus.

Moreover, as CHIKV is often co-circulating with other arthropod-borne pathogens, it is of importance to consider co-infections (e.g., ZIKV, DENV, and Plasmodium falciparum) as risk factors potentially exacerbating the clinical outcome. However, more studies are required to create a consensus on the consequences of these events.

Global vigilance and vector control measures are needed, taking into account that a large range of mosquitoes are or may become competent for viral transmission. So, those actions specifically targeting the two main vectors (i.e., *Ae aegypti* and *Ae albopictus*), would not be sufficient, and could even favor the expansion and gain of competence of other species.

Finally, while NHPs are clearly involved in enzootic transmission in Africa, very little is known about the role of animals in the maintenance of the virus in sylvatic or peri-urban cycles outside this continent. Attention should be paid to the possible establishment of natural reservoirs contrasting eradication measures.

## Figures and Tables

**Table 1:** Reported natural vertebrate animal hosts of the chikungunya virus. NHPs—non-human primates.

World Region	Species	Common Name	Method of Detection	References
	NHPs:			
Africa	*Cercopithecus aethiops*	Vervet monkey	Isolation and specific antibodies	[27,28,31]
	*Cercopithecus mitis*	Blue monkey	Specific antibodies	[67]
	*Cercopithecus ascanius*	Red tailed monkey	Specific antibodies	[67,71]
	*Galago senegalensis*	Senegal bushbaby	Isolation and specific antibodies	[27,31]
	*Papio papio*	Guinea baboon	Isolation and specific antibodies	[27,66]
	*Papio cynocephalus*	Yellow baboon	Specific antibodies	[67]
	*Papio ursinus*	Cape baboon	Specific antibodies	[28,82]
	*Erythrocebus patas*	Patas monkey	Isolation and specific antibodies	[27,66]
	*Chlorocebus sabaeus*	African green monkey	Isolation and specific antibodies	[27,66]
	*Mandrillus sphinx*	Mandrill	Specific antibodies	[70]
Indian Ocean	*Eulemur fulvus*	Brown lemur	Specific antibodies	[69]
Indian Ocean and Asia	*Macaca fascicularis*	Crab eating macaque	Isolation and specific antibodies	[74,77,78]
Asia	*Macaca nemestrina*	Pig tailed macaque	Specific antibodies	[75]
South America	*Ateles marginatus*	Spider monkey	Specific antibodies	[79]
	*Callithrix jacchus*	Common marmoset	Specific antibodies	[79]
	*Sapajus xanthosternos*	Golden-bellied capuchin	Specific antibodies	[79]
	*Sapajus robustu*	Crested capuchin	Specific antibodies	[79]
	*Sapajus flavius*	Capuchin monkey	Specific antibodies	[79]
	**Mammals:**			
Africa	*Xerus erythropus*	Palm squirrel	Isolation	[31]
	*Rattus rattus*	Ship rat	Specific antibodies	[69]
	*Scotophillus species*	Bat	Isolation	[31]
	*Syncerus caffer nanus*	Buffalo	Specific antibodies	[70]
	*Loxodonta africana*	Elephant	Specific antibodies	[70]

**Table 2:** Chikungunya virus (CHIKV) RNA detection in biological fluids in viremic patients during the Italian outbreak (2017).

Specimen	Tested	Positive
**Saliva**	5	1
**Urine**	9	1
**Ocular swabs**	2	0
**CVF**	4	1

CVF = Cervico-vaginal fluid.
